# Artifacts and Anomalies in Raman Spectroscopy: A Review on Origins and Correction Procedures

**DOI:** 10.3390/molecules29194748

**Published:** 2024-10-08

**Authors:** Ravi teja Vulchi, Volodymyr Morgunov, Rajendhar Junjuri, Thomas Bocklitz

**Affiliations:** 1 Institute of Physical Chemistry (IPC) and Abbe Center of Photonics (ACP), Friedrich Schiller University Jena, Member of the Leibniz Centre for Photonics in Infection Research (LPI), Helmholtzweg 4, 07743 Jena, Germany; ravi.vulchi@uni-jena.de (R.t.V.); volodymyr.morgunov@uni-jena.de (V.M.); rajendhar.junjuri@uni-jena.de (R.J.); 2 Leibniz Institute of Photonic Technology, Member of Leibniz Health Technologies, Member of the Leibniz Centre for Photonics in Infection Research (LPI), Albert-Einstein-Strasse 9, 07745 Jena, Germany

**Keywords:** artifacts, correction procedures, numerical methods, deep learning, Raman spectral analysis

## Abstract

Raman spectroscopy, renowned for its unique ability to provide a molecular fingerprint, is an invaluable tool in industry and academic research. However, various constraints often hinder the measurement process, leading to artifacts and anomalies that can significantly affect spectral measurements. This review begins by thoroughly discussing the origins and impacts of these artifacts and anomalies stemming from instrumental, sampling, and sample-related factors. Following this, we present a comprehensive list and categorization of the existing correction procedures, including computational, experimental, and deep learning (DL) approaches. The review concludes by identifying the limitations of current procedures and discussing recent advancements and breakthroughs. This discussion highlights the potential of these advancements and provides a clear direction for future research to enhance correction procedures in Raman spectral analysis.

## 1. Introduction

Sir C.V. Raman discovered the Raman effect in 1928, which forms the basic principle of Raman spectroscopy based on the inelastic scattering of photons [1]. However, routine Raman spectroscopic experiments were not possible until the development of the laser in the 1960s [2]. When a monochromatic light source, typically a laser, illuminates a sample, most photons are elastically scattered, termed Rayleigh scattering. However, a small fraction of the incident photons, approximately one in every $10^{6}$, interact with the vibrational modes of the molecules within the sample. This interaction leads to inelastic scattering, a process known as Raman scattering. This interaction causes the photons to either gain energy (anti-Stokes scattering) or lose energy (Stokes scattering), resulting in a shift in the energy of the scattered photons. This energy shift captures a molecular fingerprint unique to the specific chemical structure of the sample in the Raman spectrum, as it reflects the energy of existing vibrations [3]. As one of the primary techniques in vibrational spectroscopy, Raman spectroscopy delivers unique “fingerprint” vibrational spectra of analytes for straightforward characterization, making it vital in various scientific fields, such as material science, nanotechnology, and beyond [4].

Continuous advancements in Raman spectroscopy have significantly enhanced the technique’s ability to capture detailed molecular-level information. Its label-free and non-destructive nature and real-time visualization capabilities make it suitable for in vivo applications [5,6,7,8]. Despite these strengths, Raman spectroscopy has its own challenges. The inherently weak Raman signal, resulting from the non-resonant interaction of photons with molecular vibrations, is highly susceptible to various artifacts and anomalies. These issues can arise from multiple sources, including the experimental setup, sample characteristics, and sampling process [9,10,11].

For example, instrumental noise from detectors and electronic components can introduce unwanted signals, complicating data analysis [12,13,14]. Furthermore, artifacts from the sampling process, such as motion artifacts due to sample movement, can distort the spectra. These motion artifacts introduce significant noise and baseline shifts, making data analysis more complex [15,16,17]. Furthermore, while Raman spectroscopy excels in aqueous environments, making it suitable for pharmaceutical manufacturing, bioprocessing, and ophthalmic applications [18], biological samples often emit fluorescence [19]. This fluorescence, a sample-related factor, generates a background signal that can obscure the Raman signal, adding another layer of difficulty to data interpretation [19,20,21,22].

Understanding and addressing artifacts is crucial for ensuring the reliability of Raman spectroscopic analyses. The first comprehensive report on the various artifacts and anomalies in vibrational spectroscopy, particularly Raman spectroscopy, highlighted the significant impact of these artifacts on data accuracy [9]. This report discussed the early observations of instrumentally induced artifacts and the subsequent technological advancements that have mitigated some of these issues. While numerous studies have addressed specific artifacts and proposed individual correction techniques [12,15,16,23,24,25,26,27,28,29,30], a comprehensive report has yet to incorporate the wide range of common artifacts encountered in Raman spectroscopy and the limitations of current correction methods. Additionally, to our knowledge, no report has compared all the common artifacts and existing correction procedures in one article. This review aims to fill this gap by:

- Identifying and categorizing the common artifacts and anomalies in Raman spectroscopy and analyzing their underlying causes and effects on the Raman data;
- Reviewing the existing and emerging artifact correction and anomaly mitigation procedures, including experimental techniques, numerical or computational approaches, and deep learning (DL) methods;
- Discuss current correction procedure limitations and suggest future research directions.

In this review, we divide our discussion into three main parts. After the introduction, we provide an overview of the origins and types of artifacts and anomalies in Raman spectroscopy, examining instrumentation and methodological sources of artifacts and sample-related issues and environmental factors. The following section explores advanced correction techniques and strategies for mitigating artifacts and anomalies in Raman spectroscopy. This section has three subsections: numerical methods for artifact correction, experimental techniques for artifact minimization, and DL-based correction procedures. We then present existing case studies and applications demonstrating the effectiveness of these correction methods. Finally, we summarize the current limitations of correction procedures, noting that some artifacts still lack effective correction methods, and suggest future directions for research that could potentially improve Raman data analysis.

## 2. Origins and Types of Artifacts and Anomalies

**Figure 1:**
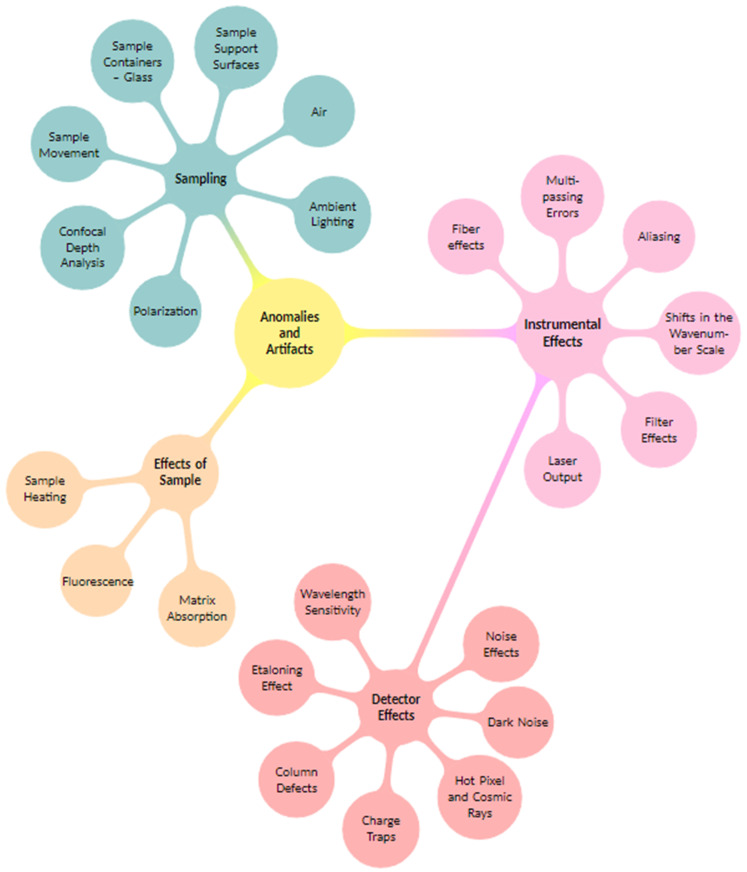
Mind map categorizing various artifacts and anomalies in Raman spectroscopy. It categorizes artifacts into three main groups: sampling, instrumental effects, and effects of sample. The instrumental effects category further branches into detector effects.

In scientific measurements, imperfections often arise in the form of artifacts and anomalies. Artifacts are features observed in an experiment that are not naturally present but occur due to the preparative or investigative procedure [31]. Also, effects that occur in parallel result in artifacts, e.g., the fluorescence baseline contribution in Raman spectra. On the other hand, anomalies are unexpected deviations from standard, regular, or expected [31]. Figure 1 displays a mind map categorizing the various types of artifacts and anomalies in Raman spectroscopy, providing a representation of their origins. Understanding these classifications helps identify, correct, and prevent these imperfections, thereby enhancing the accuracy and reliability of spectral analysis.

In the context of Raman spectroscopy, these terms refer to deviations that can distort data accuracy. Artifacts in Raman spectroscopy are typical data features introduced by the measurement process or equipment. Anomalies are unexpected variations that do not conform to standard patterns arising from sample impurities, environmental factors, or unforeseen experimental conditions. In any real system, there will be background signals due to impurities in the sample, emission from optical components, scattered light at the incident wavelength, and dark counts from the detector [32].

Researchers have extensively studied and listed various artifacts across vibrational spectroscopic techniques. For example, in mid-infrared spectroscopy, issues such as sample preparation errors and environmental interferences like atmospheric water vapor and carbon dioxide are documented [33]. In near-infrared spectrometry, the artifacts reported primarily resulted from sample handling and instrumental limitations, which affected spectral accuracy [34]. Researchers highlighted Fourier Transform Raman Spectroscopy for its sample-independent artifacts, in which advancements in detector technology have heightened sensitivity but also increased the prevalence of artifacts like etaloning, charge traps, and cosmic ray interference [35]. In the case of Tip-Enhanced Raman Spectroscopy (TERS), near-field artifacts that arise from multiple scattering events involving the tip, leading to spatial variations in Rayleigh and Raman scattering, are reported [28].

To sum up, artifacts and anomalies in Raman spectroscopy can be grouped into three main categories [9]:

- Instrumental effects introduced by measuring instruments.
- Sampling-related effects caused by the sampling process.
- Sample-induced effects that are properties or behaviors of the sample itself.

### 2.1. Instrumental Effects

**Figure 2:**
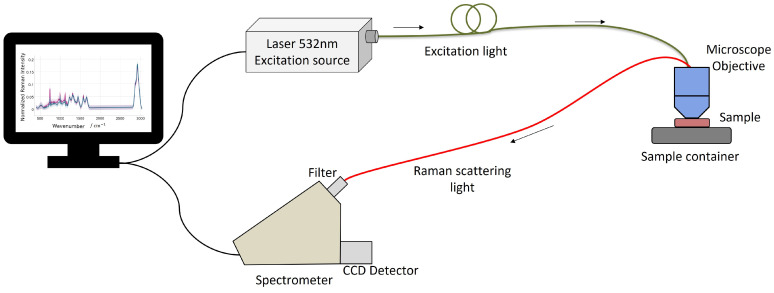
The typical layout of Raman instrumentation, which consists of the following key components: laser source, optics for sample illumination and collection, filters, spectrometer, and detector.

Various instrumental factors heavily influence the quality and accuracy of spectral data in Raman spectroscopy. The typical layout of a Raman instrumentation setup often includes a fiber-coupled laser source and spectrometer, as shown in Figure 2. This configuration allows flexibility and precision in directing the laser to the sample and collecting the scattered light. However, each component within this setup, including the laser, detector, and optical elements, plays a crucial role in shaping the resulting data. For instance, the type of detector used, such as CCD or FT detectors, can significantly influence the noise level and sensitivity of the measurements [36]. The choice of laser wavelength and its stability are also pivotal, as they can affect the degree of fluorescence interference and the quality of the spectral data [36]. Furthermore, the power delivered to the sample and the specific positioning of the sample relative to the optical path can impact the intensity and resolution of the observed spectral features. Lastly, differences in calibration and standardization across various instruments require meticulous attention to ensure consistency in Raman shift values, facilitating accurate and reliable spectral comparisons [10,37]. In this section, we delve into how each instrumental component, from the laser and detector to the sampling geometry, affects the resultant Raman data, emphasizing the importance of correction and calibration procedures to mitigate these effects.

#### 2.1.1. Laser Source

The choice of laser wavelength is critical for Raman spectroscopy, as it affects the intensity of the Raman scattering and the level of fluorescence interference. Researchers commonly use laser wavelengths of 532 nm, 785 nm, and 1064 nm, each offering different advantages depending on the sample analyzed [3,38]. The Raman signal is proportional to the amount of laser power, so more power usually produces a stronger signal. High-power, stable lasers are essential for obtaining clear and precise Raman spectra, ensuring consistent sample illumination [39].

However, all samples have a laser power density threshold beyond which structural, chemical, or non-linear effects changes may happen. For instance, laser output can introduce significant noise if it contains additional non-lasing lines. These extra lines interfere with the Raman spectrum, introducing unwanted signals. The output of lasers, such as the Nd:YAG laser, often contains additional emission lines beyond the primary lasing wavelength. For instance, while the Nd:YAG laser predominantly emits at 1.064 microns, it also produces emissions at other near-infrared wavelengths. These unwanted emissions can introduce noise and spurious peaks in the Raman spectrum if not filtered enough. Effective mitigation includes using a 1.064-micron notch filter and a 1.3-micron short pass filter to block these extraneous lines [35].

Another effect of the laser is that instabilities in laser intensity and wavelength can cause artifacts in Raman spectra. Variations in laser output can lead to noise and baseline fluctuations, obscuring the true Raman signal. Flashlamp-pumped and diode-pumped Nd:YAG lasers can emit additional non-lasing lines, which, if not adequately filtered, will result in these artifacts. Proper optical filtering using bandpass or holographic filters is crucial to remove these spurious emissions and ensure a clean Raman spectrum [9].

#### 2.1.2. Optics for Sample Illumination and Collection

Depending on the setup and specific application, the excitation light is directed onto the sample using a combination of lenses and mirrors or through optical fibers. Lenses and mirrors accurately direct and focus the laser beam onto the sample, ensuring a well-defined, concentrated excitation light spot. This configuration is advantageous in setups where precise alignment and focusing are required [39]. Optical fibers, however, provide flexibility and ease of light delivery, especially in compact or remote sampling setups. They facilitate efficient transmission of the laser light to the sample and the collection of scattered light back to the spectrometer. This approach benefits setups with limited space or those requiring the sample to be placed in a specific environment [3]. In both configurations, proper alignment and quality of the optical components are crucial for maximizing the efficiency of light collection and ensuring the clarity and precision of the Raman spectra. The collected scattered light, including the Raman scattered light, is then directed to the spectrometer for analysis.

The integration of optical fibers into Raman spectroscopy has significantly evolved. First, fiber optic probes for linear and nonlinear applications were developed, demonstrating their potential in medical diagnostics and setting the stage for multimodal optical probe design [40,41,42]. Early research highlighted the fundamental physics of stimulated Raman scattering (SRS) in fibers, showing how bend-induced wavelength-dependent losses can enhance the first Stokes intensity by suppressing higher-order Stokes signals and highlighting the interplay between fiber design and Raman signal optimization [40].

As technology advanced, fiber-based Raman spectroscopy saw the introduction of CCD detectors and diode lasers. These innovations enabled versatile sampling modes for both liquids and solids, removing the need for precise optical alignment and thus enhancing efficiency and practicality [41,43]. In clinical settings, fiber-coupled probes proved effective for non-invasive skin cancer detection, showing that reasonable variations in the fiber-bending radius had a negligible impact, thus facilitating real-time diagnostics [7,41]. Further innovations led to the development of probes capable of efficiently conducting excitation and scattered light. This step increased collection efficiency and expanded the applicability of Raman spectroscopy to challenging environments [41,44,45,46]. However, using hollow-core fibers while reducing fiber-induced Raman background presented handling challenges, particularly in liquid environments [7].

**Figure 3:**
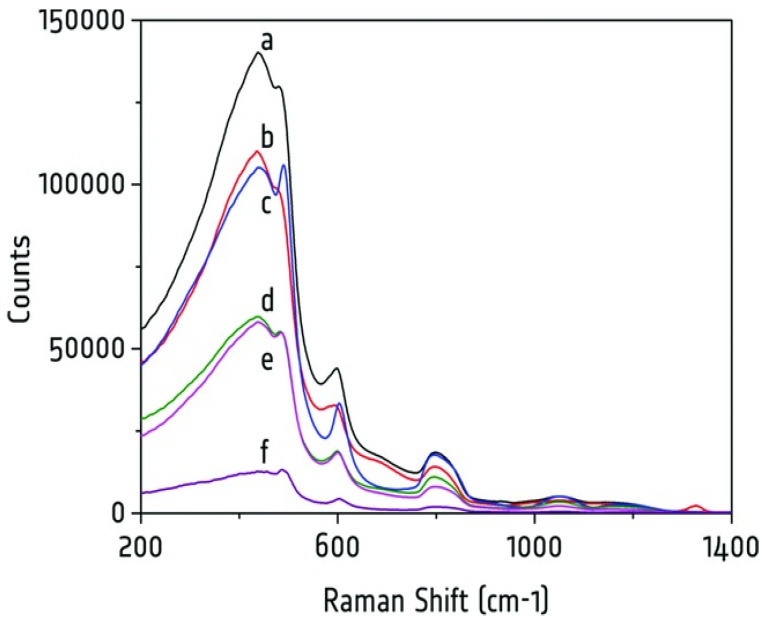
Visualizing the transmitted Raman spectra obtained from different fiber optic materials, highlighting the dependence of Raman background on fiber design. The *y*-axis represents the counts, indicating the intensity of the signal, while the *x*-axis represents the shift. The spectra labeled a through f correspond to various fiber types, demonstrating unique characteristics in terms of background noise, including different core diameters, cladding diameters, and coatings. Spectrum ‘a’ shows the highest background signal, while spectrum ‘f’ shows the lowest background signal. Other spectra (b, c, d, and e) show lower background levels. The integration time for each measurement was 200 ms, with fiber lengths, except for HC-800-02, being 1 m. This figure emphasizes the impact of the fiber material and design on the Raman background signal. Reprinted with permission [42].

However, certain artifacts are inherent in these applications due to the application of fibers. For example, the choice of fiber coating materials significantly influenced background levels. Low-OH fused-silica cores generally produced the lowest backgrounds, making material selection a critical factor in fiber design [7,43]. This was especially vital for applications like in vivo diagnostics, where minimizing background signals was essential for maximizing spectral clarity, especially in the high-wavenumber region [47]. The choice of fiber material also plays a crucial role in determining the level of Raman background noise. Figure 3 illustrates how silica core/silica cladding low-OH fibers effectively minimize Raman background signals when designed with moderate numerical apertures. By contrast, polymer-clad fibers generate significantly higher background signals, emphasizing the importance of material selection in achieving optimal performance [42].

#### 2.1.3. Filters

Filters like notch or edge filters are crucial components in Raman spectroscopy. They filter the intense Rayleigh scattered or reflected light while allowing the weaker Raman scattered light to pass through, ensuring that only the relevant Raman signal reaches the spectrometer for analysis. Filter effects also affect the quality of Raman data. Filters used in Raman spectroscopy, such as notch and band-reject filters, can degrade over time or be improperly characterized, leading to insufficient blocking of the Rayleigh line and introducing spurious signals into the Raman spectrum. Improperly maintained filters can cause spectral distortions and baseline anomalies, reducing the signal-to-noise ratio and compromising data quality [35]. The inner filter effect (IFE) also occurs when the sample’s optical density affects the Raman signal’s excitation and emission paths [48]. In surface-enhanced Raman spectroscopy (SERS), nanoparticles can either enhance or attenuate the Raman signal due to this effect, which leads to a nonlinear relationship between signal intensity and analyte concentration, causing significant errors in quantitative analysis [49].

#### 2.1.4. Spectrometer

The spectrometer disperses the filtered Raman scattered light into its constituent wavelengths using a diffraction grating. This process separates the different wavelengths of light, which correspond to various vibrational modes of the sample molecules. The dispersed light is then analyzed to produce the Raman spectrum, providing detailed information about the molecular structure and composition of the sample [3]. Many modern Raman spectrometers integrate a microscope into the system. The microscope allows precise focusing of the excitation light onto a minimal sample area and collects scattered light from the same localized region. This integration is handy for analyzing microscopic areas and obtaining high-resolution Raman spectra [50]. The microscope’s ability to target specific regions enhances its versatility and precision, making it suitable for various applications, from materials science to biological studies [3].

The width of the spectrometer slit influences both the intensity and resolution of the spectra. A wider slit increases the Raman signal intensity but decreases spectral resolution, leading to broader spectral peaks. By contrast, a narrower slit enhances resolution but reduces signal intensity [51]. The spectrometer’s overall design, including factors like optical layout and spectral efficiency, also affects the signal formation process. For instance, accurately determining the instrumental spectral response function (ISRF) or instrument transfer function (ITF) is crucial, as these factors depend on the slit function, diffraction phenomena, and optical aberrations [45,52]. Equally crucial is the overall calibration of the spectrometer, which includes factors such as spectral response and alignment. This calibration ensures accurate Raman shift values and consistent spectral output across different measurements [53]. The reliability of your spectral data is directly dependent on the proper calibration and maintenance of these spectrometer components [13,14].

Other notable instrumental effects include shifts in the wavenumber scale, aliasing, and multi-passing errors. Variations in the instrument’s limiting aperture or sample alignment can cause shifts in the wavenumber scale, leading to inaccuracies in peak positions critical for identifying molecular vibrations [9]. FT-Raman spectrometers use an internal helium-neon laser for wavenumber calibration, but misalignment can still introduce linear shifts that require correction [54].

Aliasing occurs when high-frequency components of the signal are under-sampled, causing them to appear as lower-frequency artifacts in the spectrum, leading to misinterpretation of spectral data [9]. To address this issue, a stimulated Raman loss spectrometer for metrological studies incorporates low-pass filters to remove high-frequency components before they cause aliasing [55]. Multi-passing errors occur when the signal is reflected multiple times within the spectrometer, causing distortions and inaccuracies in the recorded spectrum [9]. A low-pressure multipass Raman spectrometer effectively reduces these errors by carefully controlling the number of passes and ensuring precise alignment of optical components [56]. An advanced multiple-pass Raman spectroscopy setup enhances sensitivity and resolves multi-passing errors by precisely managing the optical path and reflective surfaces [57].

#### 2.1.5. Detector

Detectors in Raman spectroscopy play a crucial role in capturing the weak Raman scattered light and converting it into an electrical signal for analysis. Initially, Raman spectroscopy used photomultiplier tubes (PMTs) as detectors. PMTs are highly sensitive and capable of detecting low-intensity signals. However, they have limitations in spatial resolution and the ability to capture a broad range of wavelengths simultaneously. With the introduction of charge-coupled device (CCD) in the 1970s and 1980s, the exit slit of the spectrometer was replaced by a linear CCD detector, enabling the acquisition of many channels at the same time. The CCD detector converts the dispersed light into an electronic signal by capturing the light photons and converting them into electrical charges. This conversion process, which involves accumulating charge proportional to the light intensity in each pixel of the CCD, is essential for processing the light into a digital format that can be analyzed to generate the Raman spectrum, revealing the sample’s molecular fingerprint. Advanced detectors, such as intensified CCDs (ICCDs) and electron-multiplying CCDs (EMCCDs), have further enhanced detection capabilities by amplifying the signal and boosting sensitivity, making them particularly useful in low-light conditions and for single-molecule detection. [3,39].

In Raman spectroscopy, the detector’s choice and performance significantly impact the accuracy and quality of the obtained spectra. Researchers widely use CCDs due to their high sensitivity and low noise characteristics, which enhance the system’s quantum efficiency [58]. However, this increased sensitivity also amplifies certain artifacts, such as etaloning and fixed pattern noise (FPN) caused by interference within the detector substrate [58,59]. FPN is inherent in imaging sensors, including CCDs [60]. This systematic noise remains constant across multiple images or spectra, caused by non-uniformities in a CCD detector’s pixel response and readout electronics [9].

FPN arises from non-uniformities in the detector, leading to variations in pixel response that create a consistent pattern across multiple measurements. This noise can mask spectral features, especially in weak Raman signals [59,61]. In back-illuminated CCDs, thinning the detector substrate to enhance sensitivity further introduces irregularities, aggravating FPN effects. The resultant fixed pattern can obscure true Raman peaks by overlaying a consistent noise pattern on the spectra, making distinguishing between true spectral features and artifacts challenging [59].

**Figure 4:**
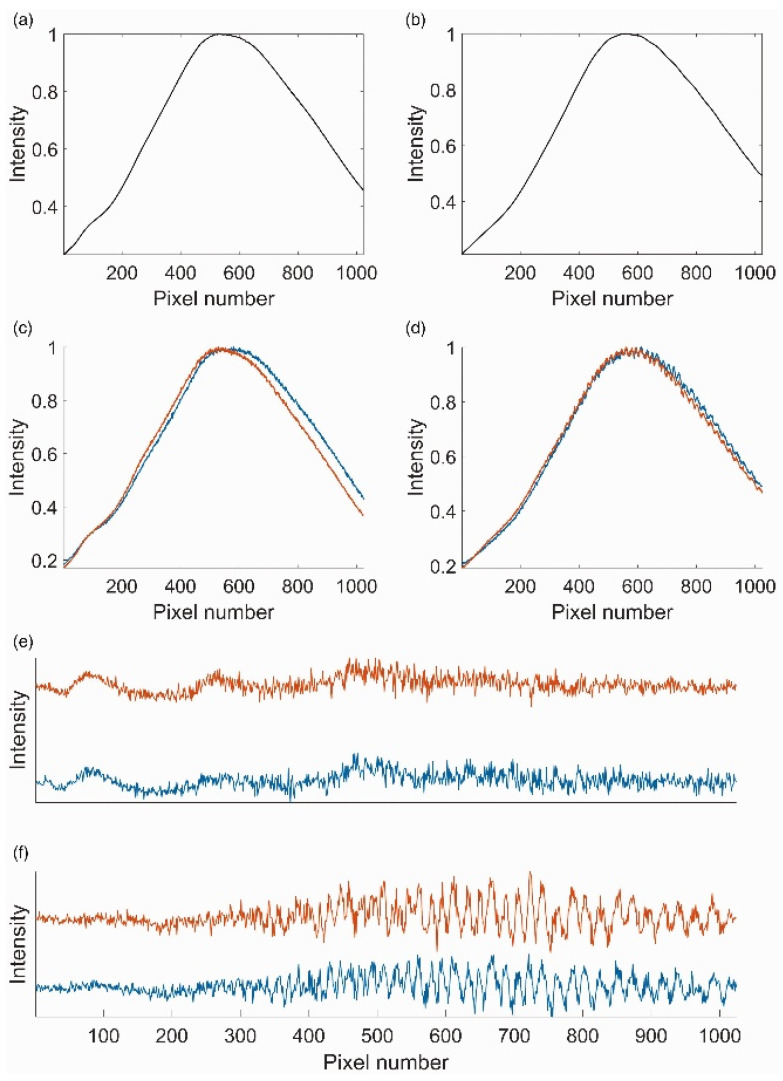
Visualizing the impact of FPN and etaloning effects on Raman spectra. Panels A and B display the averaged spectra over all rows of data collected on CCD1 (**a**) and CCD2 (**b**), showing no visible fixed pattern. Panels C and D show individual neighboring rows from CCD1 (**c**) and CCD2 (**d**), highlighting the presence of etalon fringes more prominently in CCD2. Panels (**e**,**f**) present high-pass filtered spectra from individual rows of CCD1 (**e**) and CCD2 (**f**), emphasizing the wavelength-dependent oscillations due to etaloning and variations in pixel response due to FPN. These figures demonstrate the impact of FPN and etaloning on Raman spectra, with CCD2 showing more artifacts than CCD1 and etaloning effects on Raman spectra. Reprinted with permission [59].

Etaloning, a specific type of FPN, occurs due to multiple internal reflections within the thin substrate of back-illuminated CCDs [62]. The interference causes wavelength-dependent oscillations in the detected signal, called fringes [8], which are depicted in panels C and D of Figure 4. The etalon effect becomes particularly pronounced in the near-infrared (NIR) region, where the lower absorption coefficient allows more light to reflect within the detector, amplifying the interference patterns. These sinusoidal variations are visible in panels C and D of Figure 4 and can significantly distort the Raman spectra, especially when the Raman signals are weak and superimposed on strong fluorescence backgrounds [59]. Panels E and F further emphasize these distortions, showing high-pass filtered spectra from CCD1 and CCD2, respectively, where the wavelength-dependent oscillations due to etaloning are more evident.

This distortion mimics or obscures Raman peaks, complicating data interpretation and potentially leading to inaccuracies in spectral analysis [8]. In practical applications, such as in vivo Raman spectroscopy, ambient light and high-fluorescence backgrounds further exacerbate the etalon effect. The additional light sources introduce spectral contamination, which, combined with the etalon fringes, results in more pronounced distortions that are difficult to correct without introducing further artifacts [8].

**Figure 5:**
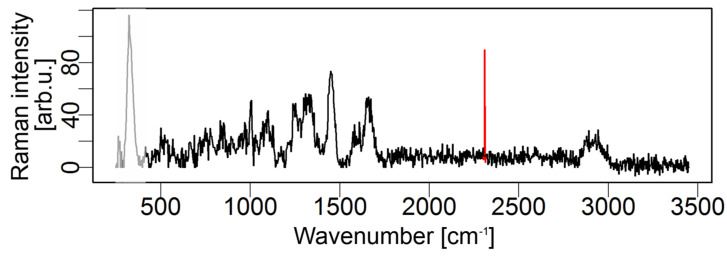
A Raman spectrum with a clear cosmic ray spike (highlighted in red) at around 2400 ${cm}^{-1}$. The spike is an artifact caused by high-energy cosmic particles interacting with the CCD detector, resulting in a sharp, intense peak not representative of the sample composition. Reprinted with permission [24].

Additionally, CCDs are prone to cosmic ray artifacts, which appear as sharp, spurious peaks in the spectra and can significantly affect data quality if not adequately mitigated, as shown in Figure 5 [63,64,65]. Cosmic rays are high-energy particles from space that can interact with the CCD detectors, producing sharp, unidirectional spikes in the Raman spectra [64]. These spikes can obscure spectral features and complicate data interpretation if not correctly removed, highlighting the need for spike removal algorithms and data pre-processing.

### 2.2. Sample-Related Artifacts

In principle, the non-resonant Raman spectrum of a sample should remain consistent regardless of the laser wavelength. However, different wavelengths can cause significant variations in the spectra due to numerous sample-related factors. In the previous section, we examined the artifacts contingent on the type of instruments used for the measurement. In this section, we will delve into the sample-dependent factors that may cause Raman spectra to vary with the wavelength of the excitation laser. These sample-related artifacts arise from the interactions between the sample and the incident laser light, the sample environment, and the intrinsic properties of the sample [9].

For example, laser-induced sample heating causes thermal emission, a rising background superimposed on the Raman spectrum [66]. This effect can significantly degrade the quality of the Raman spectra, particularly at Raman shifts from about 2000 ${cm}^{-1}$ to the detector cut-off at 3550 ${cm}^{-1}$. This thermal emission indicates substantial sample heating, which can damage thermally sensitive biomolecules like proteins and enzymes [67]. Also, increasing laser power to enhance Raman signal intensity can lead to an increased baseline due to thermal emission, altering the relative intensities of Raman bands. High laser power can sometimes alter the sample’s structure due to heating, potentially modifying its composition or crystal structure. It was shown that even low laser powers could raise the sample temperature by 40–50 K, with damage occurring at laser energies as low as 300 mW, leading to temperatures around 400 K [9,67].

**Figure 6:**
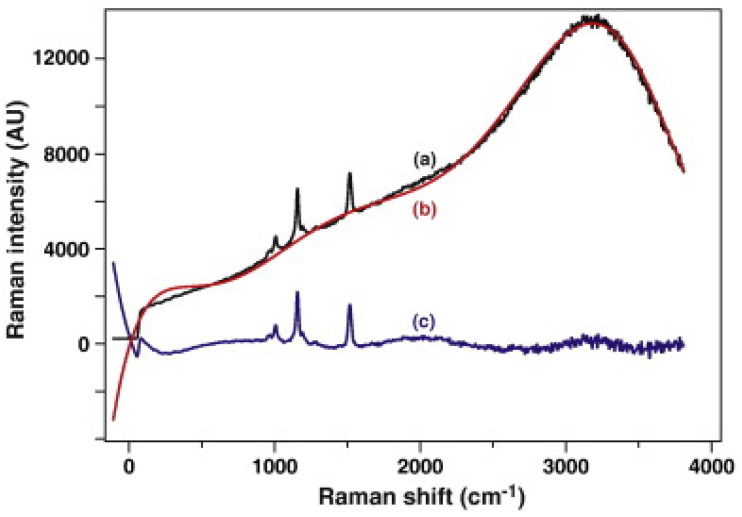
Visualizing the removal of the fluorescence background in confocal Raman microspectroscopy. (a) The raw Raman spectrum (black) shows significant fluorescence interference. (b) The researchers used a polynomial baseline to model the fluorescence (red). (c) The corrected spectrum (violet) demonstrates the removal of the fluorescence background, revealing the true Raman signal. Reprinted with permission [21].

Also, fluorescence is the most commonly encountered issue in Raman spectra due to shorter laser wavelength, leading to a high background signal that masks the desired Raman signals, as shown in Figure 6 [9]. Fluorescence arises when molecules absorb photons and re-emit them at a longer wavelength. This phenomenon can significantly interfere with Raman scattering, especially since fluorescence intensity is typically several orders of magnitude higher than Raman scattering signals [20]. The prevalence of fluorescence in Raman spectroscopy is primarily due to the overlap of excitation wavelengths with the absorption bands of fluorophores present in many samples. Visible light, typically used in Raman spectroscopy, excites these fluorophores, leading to significant fluorescence emission. This overlap is noticeable when using visible light for excitation, which often excites fluorescence in organic compounds and biological samples [21].

Additionally, fluorescence interference can complicate the quantitative analysis of Raman spectra. Fluorescence emits a broadband signal in the same wavelength interval as the Raman signal. This emission can be several orders of magnitude more intense than the Raman signal, creating a substantial background that can obscure the Raman peaks [32]. The fluorescence background can sometimes intensify to $10^{6}$ to $10^{8}$ times more than the Raman scattering, completely masking the Raman signal [22].

### 2.3. Artifacts from the Sampling Process

Sampling artifacts occur due to various factors during the collection of Raman spectral data, significantly affecting the accuracy and reliability of the results. These artifacts can originate from environmental factors, sample handling, and specific characteristics of the instrumentation used [9]. Below, we explore the primary sampling artifacts.

The materials used for sample containers can significantly contribute to artifacts in Raman spectroscopy. Glass containers, for example, can introduce broad peaks related to silica, complicating the spectral analysis [45]. The container’s thickness, curvature, and cleanliness further influence Raman measurements by altering the path or intensity of the laser light, leading to poorly recorded spectra. Traditional Raman spectroscopy frequently encounters challenges with containers that exhibit inherent fluorescent properties or significant Raman scattering characteristics, such as colored glass. In these instances, fluorescence or scattering can obscure the contents of Raman signals, leading to potentially inaccurate or unclear spectral data. Laser light interacting with container materials can complicate Raman spectra, especially when light scattering from opaque materials combines with fluorescence from colored glass containers like green or amber glass, masking the sample’s signals [68].

**Figure 7:**
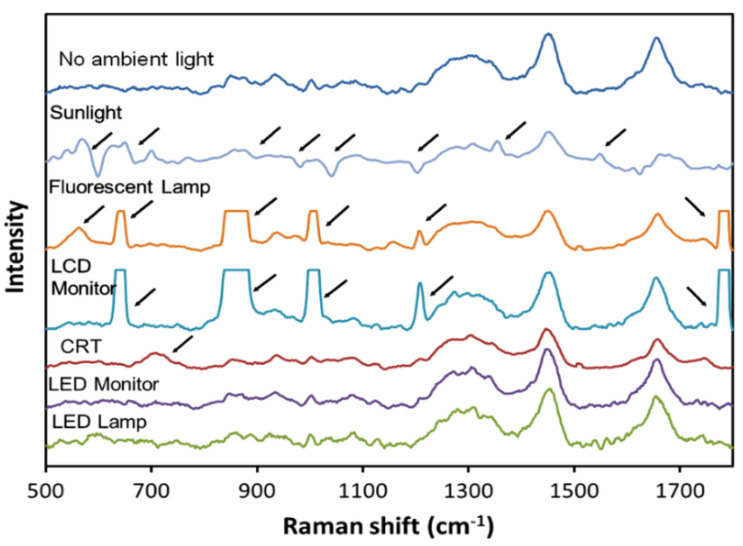
Visualizing the impact of different ambient light sources on the Raman spectra of human palm skin. The figure displays spectra with and without ambient light from indirect sunlight, fluorescence lamps, LCD monitors, CRT monitors, LED monitors, and LED lamps. Arrows indicate spurious peaks and distortions in the spectra caused by these light sources, highlighting the challenges ambient lighting can pose to the integrity of Raman spectral data. These effects underscore the necessity for effective filtering techniques to isolate the true Raman signals from ambient light interference in clinical and field settings. Reprinted with permission [69].

In addition to container-induced artifacts, ambient light can significantly impact Raman spectral data. Ambient light, particularly from NIR sources, can introduce unwanted spectral features that mask the true Raman signals. Techniques such as using narrow band-pass filters to block ambient NIR light selectively are essential to ensure the integrity of Raman measurements in environments with variable lighting conditions, such as clinical settings or in-field analysis [69,70]. Figure 7 demonstrates these effects with differences in the spectra obtained under various types of lighting, underlining the necessity of controlling ambient light during measurements.

## 3. Strategies for Mitigating Artifacts and Anomalies in Raman Spectroscopy

Effective mitigation of artifacts and anomalies in Raman spectroscopy relies on numerical methods, experimental techniques, and deep learning-based correction procedures. Studies have outlined comprehensive workflows for preprocessing Raman spectra to remove corrupting effects and extract subtle spectral variations of interest [65,71,72]. Central to these workflows is chemometrics, which involves applying mathematical and statistical techniques to design experiments, process data, and interpret complex information.

Chemometrics encompasses a variety of procedures. These include experimental design to optimize measurement conditions, data preprocessing techniques such as baseline correction, noise removal, and normalization, as well as statistical learning methods like clustering and regression to interpret the processed data. This range of techniques demonstrates the extended nature of Raman spectral analysis. [65].

This section covers correction procedures for the first two phases of artifact correction: pre-treatment and preprocessing. Section 3.2 and Section 3.3 include detailed tables for computational methods and deep learning-based correction procedures. Table 1 summarizes the correction procedures for these phases following the protocol and outlines a systematic workflow beginning with cosmic spike removal, followed by baseline correction, smoothing, and normalization [65]. Table 2 summarizes algorithms used to correct multiple artifacts with a single approach for DL-based methods, highlighting how autoencoders, 1D-CNNs, and LSTM networks are applied to spectral correction. Unlike Table 1, Table 2 does not separate artifacts by algorithm because DL techniques tend to address multiple artifacts simultaneously using the same models.

### 3.1. Experimental Design for Artifact Minimization

Proper experimental design involves carefully considering various factors, including the choice and modifications to the experimental setup, calibration, maintenance procedures, sample preparation, and environmental control. This section outlines the currently employed key aspects of experimental design that aim to minimize artifacts and improve the quality of Raman spectroscopic data. Design of Experiments (DOE) is a systematic method to determine the relationship between factors affecting a process and its output. In Raman spectroscopy, DOE helps minimize artifacts by optimizing experimental conditions to reduce variability and improve data accuracy. From a chemometrics perspective, DOE mainly consists of the measurement protocol and sample size planning (SSP) [65].

The measurement protocol is a cornerstone of experimental design and outlines detailed procedures for sample preparation and the specific instruments to be used. Proper measurement protocols ensure the experimental setup is consistently optimized to minimize artifacts. This includes choosing the correct or shifting excitation wavelengths, such as near-infrared lasers for highly fluorescent samples, which helps to reduce fluorescence interference [73]. Proper measurement protocols optimize the experimental setup to minimize artifacts [50].

For example, Shifted Excitation Raman Difference Spectroscopy (SERDS) [74] and Wavelength-Modulated Raman Spectroscopy (WMRS) [75] are spectral acquisition protocols that minimize fluorescence artifacts. Both are classified as wavelength-domain methods, exploiting the difference in response to excitation wavelength changes between Raman and fluorescence signals to suppress fluorescence [20,76]. Similarly, using a shorter wavelength excitation can reduce the risk of the etaloning effect [8,77].

While the choice of instruments in Raman spectroscopy is often dictated by what is available in the lab, thoughtful consideration of key factors can significantly help reduce a few artifacts. A NIR laser source, like a distributed feedback (DFB) diode laser emitting at 785 nm, effectively reduces fluorescence interference. When paired with SERDS, it minimizes fluorescence. UV light also suppresses fluorescence, provided the sample can withstand potential phototoxicity. [65,78,79]. Spatially Offset Raman Spectroscopy (SORS) significantly enhances the ability to identify materials through challenging packaging, such as translucent plastics and colored glass, by reducing container interference [68]. Additionally, using the confocal light collection and tips with smooth or transparent shafts helps remove near-field artifacts caused by multiple scattering events, ensuring more accurate Raman data at the nanoscale [28].

Also, selecting the appropriate detector material is essential in reducing fluorescence interference. For example, using an indium gallium arsenide (InGaAs) detector with a longer incident wavelength (993 nm) significantly minimizes fluorescence compared with a silicon-based CCD detector at shorter wavelengths (903 nm) [80].

Regular calibration with well-defined standards maintains the consistency and comparability of Raman spectra over time and across different instruments [10,81]. Using NIST Standard Reference Materials (SRMs) for relative intensity correction ensures consistency and reproducibility across different Raman spectrometers [73]. Calibration with appropriate wavenumber and intensity standards helps reduce artifacts by ensuring that Raman signals are well-defined and separated [53,82].

For wavenumber calibration, the standards should have well-defined and distinct Raman bands, with multiple calibration lines necessary for dispersive Raman setups to cover the entire wavenumber range of interest. Intensity standards must be homogeneous and consistently emit within the relevant wavenumber range [65]. Intensity calibration should be performed under the same conditions and optical geometry as the samples to reduce unexpected Raman peaks introduced by specific doping processes [83]. Regular calibration with these standards, especially after any changes to the instrument, mitigates various artifacts, ensuring accurate spectral data. The available standard materials for both wavenumber and intensity calibration can be found in the literature, aiding in selecting the most appropriate standards [84,85].

### 3.2. Computational Methods for Artifact Correction

Correcting artifacts in Raman spectroscopy fundamentally relies on numerical methods, which may incorporate mathematical algorithms or involve solving partial differential equations (PDEs). The most common numerical methods actively remove cosmic spikes, correct baseline drifts, eliminate fluorescence interference, and reduce noise—addressing primary artifacts that can obscure the true Raman signal. Depending on the data, preprocessing was unique for all Raman spectra [82]. The standard protocols start with removing cosmic spikes. These procedures typically include quality control to identify and remove outliers; spike removal to eliminate cosmic spikes; wavelength, wavenumber, and intensity calibration to ensure spectral accuracy; baseline correction to remove fluorescence and other background signals; smoothing to reduce noise; and normalization to standardize the spectra [65,86].

Fundamental preprocessing techniques include polynomial fitting or derivative-based methods, standard normal variate (SNV) or multiplicative signal correction (MSC), Principal Component Analysis (PCA), and Partial Least Squares Regression (PLSR). It is recommended that baseline correction be performed before normalization to handle both additive and multiplicative effects in the data effectively [87]. Additionally, selecting appropriate preprocessing methods is crucial, as their effectiveness can vary significantly depending on the characteristics of the specific dataset [88]. The preprocessing process falls into three phases: pre-treatment, preprocessing, and processing/model generation [11,81,89,90]. This discussion focuses on the first two phases, which contain the correction procedures. Table 1 summarizes existing correction procedures from the literature for each phase.

**Table 1:** Overview of established computational methods for correcting common artifacts during Raman spectroscopy’s pre-treatment and preprocessing phases. Each technique targets specific artifacts such as cosmic rays, baseline drift caused by fluorescence, and noise from the instrument. The table categorizes these artifacts based on their source (e.g., detector and fluorescence), lists the algorithms applied for correction, and concisely describes how each algorithm functions. This structure follows the standard protocol outlined by [65], which organizes the correction process stepwise.

Pre-Treatment Phase		
Artifacts (Source)	Algorithms Used	Description
**Cosmic rays (Detector)**	Upper bound spectrum method [30,91,92,93]	Removes spikes by comparing their intensity to an upper bound limit derived from multiple spectra.
	Wavelet transforms [94,95,96]	Remove spikes by filtering high-frequency noise.
	Moving window filtering [97]	By averaging within a moving window and replacing spikes with the averaged values.
	Polynomial filters [98,99,100]	Fitting polynomials by replacing high-intensity outliers with interpolated values.
	Multistage spike recognition [101,102]	Tracks change in the time domain through a multistage process, automatically identifying and confirming spikes.
	Normalized covariance, Nearest neighbor comparison (NNC) [23]	Combines single and double acquisition techniques, using normalized covariance to remove contaminated pixels.
	PCA and NNC [26,88,103,104]	Replacing contaminated spectra with best-fit spectra using NNC.
	Laplacian operator and Median filtering [24,105]	Tracks intensity changes using a Laplacian operator, applies an automated threshold to separate spikes from spectral features, and uses median filtering for interpolation.
	Moving average filter [106]	Uses modified Z-scores (standard scores adjusted for outliers) to detect spikes.
**Wavelength calibration** **(Instrument)**	Polynomial fitting [107,108,109,110]	Involves using a polynomial function to correlate pixel positions in Raman spectra to accurate wavenumbers.
	Optimization fitting algorithm [111]	Analytical calibration model based on the Czerny–Turner optical system, using grating equation and geometric optics principles
	Physical model with a fast search algorithm [112,113]	Physical model to relate pixel positions to wavelengths refined by a brute-force search and linear regression to calibrate spectral data accurately.
**Intensity calibration** **(Instrument)**	Polynomial curve fitting, cross-relation analysis [114]	Fitting a polynomial curve to remove broad signal components.
***Pre-processing Phase***		
**Fluorescence (Sample)**	Fourier transform filtering [22,115]	Broad fluorescence components are separated and filtered by converting to the frequency domain.
	Polynomial fitting [6,116,117,118,119,120,121]	The fluorescence spectrum is fitted to a low-order polynomial and then subtracted from the Raman spectrum.
	Wavelet transforms [122,123,124,125]	Low-frequency components corresponding to the fluorescence background are removed.
	First and second-order derivatives [126,127,128]	Derivative of a measured Raman spectrum reduces the magnitude of the fluorescence background.
	PCA [129,130]	Decomposes mixed spectra into principal components, isolating the Raman signal in the subsequent components.
	MSC methods [27,87,131,132,133,134,135]	Normalize spectral data by correcting for baseline offsets and scatter effects to ensure sample consistency.
	Least squares methods [136,137,138,139,140,141]	Fit a smooth baseline by minimizing a penalized least squares function, asymmetrically weighting deviations to keep the baseline below signal peaks.
	Spline smoothing method [142]	Fits a smooth baseline for Raman spectra using penalized spline smoothing and vector transformation.
	Automatic Two-side Exponential Baseline algorithm [143]	Applies two-sided exponential smoothing, which iteratively smooths the signal using exponentially decreasing weights.
	Iterative reweighted quantile regression [144]	Applies quantile regression iteratively with reweighted residuals, allowing it to distinguish between baseline and signal peaks.
	Genetic algorithm (GA) [145,146]	Based on natural selection and evolution principles, GA optimizes the parameters of methods like ALS, spline smoothing, and polynomial fitting.
	Goldindec approach [147]	Introduces the asymmetric index function, which increases the cost when the fitting curve deviates from the true baseline.
	Morphological and histogram-based baseline determination [148,149,150]	Fits a smooth baseline by using structural elements to remove unwanted features and adjusting the histogram to minimize deviations.
	Savitzky–Golay (SG)-based iterative smoothing [29,151]	Identifies Raman peaks using a negative relaxation factor and then iteratively applies Savitzky–Golay smoothing to subtract the fluorescence baseline.
	Wavelet transformation and penalized least squares fitting [152]	Detects peak positions using a wavelet transform estimates peak width through SNR enhancement and fits the background using penalized least squares.
	Iterative Smoothing-Splines with Root Error Adjustment [153]	Iteratively fitting smoothing splines to the raw spectrum, adjusting prediction errors using a root transformation to estimate and correct the baseline.
**Noise** **(Instrument, Sampling)**	Conventional scale correlation [125]	Applies quantile regression iteratively with reweighted residuals, allowing it to distinguish between baseline and signal peaks.
	Tikhonov regularization [154]	Tikhonov regularization and morphological operations in a unified variation model iteratively smooth the spectrum.
	Third-order discrete wavelet transform [25]	Decomposes the signal into different frequency components, applying a threshold to remove high-frequency noise.
	Hilbert vibration decomposition (HVD) [155]	Denoises signals by iteratively decomposing them into components based on instantaneous frequency and amplitude, extracting and compensating peaks.

While effective, classical spectral preprocessing methods have limitations. The study of baseline removal techniques identifies these limitations. These methods, such as SG smoothing and ALS, require specific parameter settings (e.g., polynomial order and smoothing window size) to be effective [115,156]. Their specificity and the need for parameter optimization for each new data set restrict their applicability. Each algorithm can only handle a specific interference, and a series of methods can only correct a specific data set of the training samples with their optimized parameters. To enhance the spectra collected from different samples with diverse characteristics and interferences, newly optimized parameters are needed to preprocess each data set. This highlights the need for methods to correct all interferences in a single step.

### 3.3. Deep Learning-Based Correction Procedures

Deep learning (DL), a subset of machine learning (ML), has garnered significant attention in Raman spectroscopy research in recent years [157,158,159,160,161]. Early research in this field includes ML methods such as support vector machines (SVM), naive Bayes, k-nearest neighbors (KNN), random forest (RF), and artificial neural networks (ANN) [162,163,164,165]. While laying the groundwork for more sophisticated approaches, these methods had their limitations. They initially improved noise reduction and baseline correction but often struggled with the high dimensionality and complex, non-linear relationships inherent in Raman spectral data [166].

Recent advancements in computational power and algorithms have significantly enhanced the capabilities of refined DL models like convolutional neural networks (CNNs) and recurrent neural networks (RNNs). These DL techniques have further improved the preprocessing of Raman spectroscopy data. Studies have shown that a single CNN model effectively removes all interferences from multiple Raman spectra data sets. When applied to Raman spectra from food and wastewater samples, CNN models improved performance by 91.4% in RMSE and 94.5% in SNR, effectively removing noise, baselines, and cosmic rays in one step [160,161]. Table 2 lists existing correction procedures from the literature on each phase.

Further, physics-informed neural networks (PINNs) have received much attention as a breakthrough in solving PDEs using neural networks [167,168]. PINNs produce more interpretable models by integrating physical principles, enhancing artifact correction, and spectral analysis. This breakthrough has sparked a new wave of interest in spectral analysis. Recently, a PINN architecture for removing background noise utilizes a loss function that includes a reconstruction error term to minimize the difference between the measured and predicted spectra and a regularization term to penalize large gradients in the predicted background spectrum [169]. In synthetic cases, the PINN model showed an R-squared value greater than 0.99, accurately reconstructing spectra under varying background noise conditions. A strong correlation was maintained on the experimental spectra of riboflavin samples, with an average concentration prediction error of around 10%. This approach ensures the smoothness of the background noise, reflecting the physical expectation that real backgrounds are typically smooth.

**Table 2:** Deep learning-based algorithms that have successfully applied to correct artifacts in Raman spectroscopy data. The table outlines the DL models, such as autoencoders, 1D-CNNs, and LSTM networks. It also briefly explains each model’s functionality and how they learn to reconstruct clean spectra or detect patterns in noisy data.

Algorithms Used	Description
Neural net comprising encoder and decoder [170]	Autoencoder learns to reconstruct clean spectra from noisy input by encoding essential features into a lower-dimensional space and decoding them back to remove noise and artifacts.
1D-CNN [171,172,173,174,175,176,177]	Convolutional filters detect and learn peaks and patterns
Long short-term memory (LSTM) [178]	Learning from environmental correlates and temporal patterns to accurately predict and adjust for measurement errors.
CNN with a custom loss function [179]	Custom loss function combines mean squared error with an additional term for peak preservation, balancing overall denoising with the retention of critical spectral features.
Convolution autoencoders [170,180]	Use convolutional layers and a comparison function to capture and correct baseline features accurately.
1D-Unet [181]	Learns using an encoder–decoder architecture in which the 1D CNNs in the encoder extract hierarchical features and the decoder reconstructs the signal.
Multi-Scale Feature Extraction Denoising (MFED) [182]	Improves the signal-to-noise ratio of spectra by using a CNN with multi-scale feature extraction and data augmentation.
ResNet and Unet [183,184,185]	Utilize residual connections to prevent the vanishing gradient problem, effectively allowing the network to learn complex representations from deep layers.
PINN [169]	The physics of light-matter interactions constrain the loss function with terms that account for the spectral contributions from the element concentration, background noise, and the continuity of the background spectrum.

## 4. Future Directions

This paper aims to draw attention to a crucial aspect of Raman spectroscopy: the fragility of the Raman scattering process. It is significantly weaker than other optical processes, and various sources can cause artifacts in the Raman spectra. Significant limitations remain despite the development of numerous advanced methods and experimental protocols to mitigate these artifacts. Experimental techniques are highly dependent on the availability and precision of instruments in the laboratory, which can vary widely across different settings. Numerical methods often need help with complex problems for which deriving a simple fitting equation, such as a polynomial or specific parameter, is difficult. Additionally, DL methods based on supervised learning require labeled data for training, which is only sometimes feasible to obtain. In situations where controlled experiments with known outcomes are impractical, correction procedures must rely on theoretical assumptions based on the physics of the phenomenon to develop acceptable correction functions.

Furthermore, there is a need for more discussion on correction procedures for specific issues, such as removing etaloning effects from Raman spectra and addressing the impact of optical fibers on the spectra. These areas still need to be explored and present significant opportunities for future research to enhance the accuracy and reliability of Raman spectral analysis. Investigating inverse modeling through PINNs and exploring unsupervised or semi-supervised DL methods are particularly promising avenues for addressing these challenges. These approaches, which leverage physical laws and reduce the dependency on labeled data, could offer robust solutions. Continued research in these areas is essential for developing more effective correction procedures for Raman spectra analysis.

## 5. Summary

The transition from traditional statistical methods to advanced techniques like DL has marked a significant shift in Raman spectra data analysis. Initially, statistical methods such as polynomial fitting were employed to correct simple artifacts like noise and baseline drifts. However, these approaches often fell short when dealing with more complex, non-linear interferences. Introducing 1D CNNs has transformed the field by automatically learning and correcting tough patterns and effectively removing all interferences across multiple Raman spectra data sets rather than addressing just one artifact at a time. Despite this progress, there is still a need for more effective correction procedures, particularly for addressing complex issues like etaloning effects and the impact of optical fibers. Continued research and the development of innovative approaches, such as PINNs that incorporate physical laws into neural network models, are crucial for overcoming these limitations and advancing the accuracy and reliability of Raman spectral analysis.
